# Special Notice

**Published:** 1876-12

**Authors:** 


☞ *Special Notice.*—Renewals are coming in daily. Thus far we can say but few discontinuances have been ordered for the ensuing year, and not one upon the ground of dissatisfaction with the journal. The pressure of the times is the invariable plea. As these discontinuances were ordered before the reduction of the terms to $2.00, we hope that these parties will reconsider their action, and renew upon our new basis. Friends, to meet the exigency of the times, we have decided upon a cheaper journal, and we shall labor to condense into a smaller compass an equal amount of matter for the benefit of our readers —so that any one who, from motives of economy, decides upon taking but one journal, will find it to his interest to take the RECORD as the journal which, at a low figure, will contain the greatest amount of practical information.

The time of our new issue will be as usual, on the 20th of the month, but we will endeavor to get out the January number somewhat sooner.

Parties desiring to renew, or to take the RECORD for the next year, please inform us at the earliest possible moment. The subscription price of $2.00 may be sent with the renewal notice, or immediately after the reception of the January number.

And let no subscriber forget to use his influence in extending our circulation.

☞Amid the multiplicity of duties that attach to the editorial and business conduct of a journal, oversights and mistakes sometimes occur. They have not been intentional with us. If, in the matter of sending receipts, we have failed to respond, please notify us, and we will make it right.

